# The M. Powell Lawton Award Lecture: The Person-Environment Fit Framework, Older Adults and Technology Interactions

**DOI:** 10.1093/geroni/igab046.2127

**Published:** 2021-12-17

**Authors:** Sara Czaja

**Affiliations:** Weill Cornell Medicine/Center on Aging and Behavioral Research, New York, New York, United States

## Abstract

M. Powell Lawton made significant contributions throughout his illustrious career to improve the quality of life of older adults. His landmark theory of person-environment fit (P-E Fit) recognized the importance of understanding the dynamic interactions between older adults and their physical and social environments and the subsequent impact of these interactions on independent living. In today’s living environments, technology is ubiquitous and can serve as both a barrier and facilitator to the ability of older people to live independently. This presentation will discuss how the P-F Fit Model can be used to clarify potential mismatches between technology systems and the characteristics, abilities, and preferences of older adult and how it can be used to guide design and training interventions to maximize the ability of aging adults to interact successfully with technology systems. Examples will be drawn from the Center for Research and Education on Aging and Technology Enhancement (CREATE) in the domains of social engagement, work, and health from technology design and intervention perspectives. The CREATE conceptual framework, consistent with the P-E Fit Model posits that users have varying needs, abilities, and attitudes; technology systems and tasks vary in demands; social, physical, and policy environments influence a person’s access to and support for technology transactions; and human-technology interactions are dynamic. A focus of the presentation will be on how a user-centered design approach is compatible with the P-E Fit model and can optimize the fit between older adults and technology systems.

